# Modifications of Grapevine Berry Composition Induced by Main Viral and Fungal Pathogens in a Climate Change Scenario

**DOI:** 10.3389/fpls.2021.717223

**Published:** 2021-12-08

**Authors:** Markus Rienth, Nicolas Vigneron, Robert P. Walker, Simone Diego Castellarin, Crystal Sweetman, Crista A. Burbidge, Claudio Bonghi, Franco Famiani, Philippe Darriet

**Affiliations:** ^1^Changins College for Viticulture and Oenology, University of Sciences and Art Western Switzerland, Nyon, Switzerland; ^2^Dipartimento di Scienze Agrarie, Alimentari e Ambientali, Università degli Studi di Perugia, Perugia, Italy; ^3^Wine Research Centre, Faculty of Land and Food Systems, The University of British Columbia, Vancouver, BC, Canada; ^4^College of Science & Engineering, Flinders University, Bedford Park, SA, Australia; ^5^School of Agriculture and Food, Commonwealth Scientific and Industrial Research Organization (CSIRO), Glen Osmond, SA, Australia; ^6^Department of Agronomy, Food, Natural Resources, Animals and Environment, University of Padova Agripolis, Legnaro, Italy; ^7^Univ. Bordeaux, Unité de recherche Œnologie EA 4577, USC 1366 INRAE, Institut des Sciences de la Vigne et du Vin, Villenave d’Ornon, France

**Keywords:** grapevine, biotic stress, *Plasmopara viticola*, leafroll virus, fanleaf virus, *Erysiphe necator*, *Botrytis cinerea*

## Abstract

The grapevine is subject to high number of fungal and viral diseases, which are responsible for important economic losses in the global wine sector every year. These pathogens deteriorate grapevine berry quality either directly *via* the modulation of fruit metabolic pathways and the production of endogenous compounds associated with bad taste and/or flavor, or indirectly *via* their impact on vine physiology. The most common and devastating fungal diseases in viticulture are gray mold, downy mildew (DM), and powdery mildew (PM), caused, respectively by *Botrytis cinerea*, *Plasmopara viticola*, and *Erysiphe necator*. Whereas *B. cinerea* mainly infects and deteriorates the ripening fruit directly, deteriorations by DM and PM are mostly indirect *via* a reduction of photosynthetic leaf area. Nevertheless, mildews can also infect berries at certain developmental stages and directly alter fruit quality *via* the biosynthesis of unpleasant flavor compounds that impair ultimate wine quality. The grapevine is furthermore host of a wide range of viruses that reduce vine longevity, productivity and berry quality in different ways. The most widespread virus-related diseases, that are known nowadays, are Grapevine Leafroll Disease (GLRD), Grapevine Fanleaf Disease (GFLD), and the more recently characterized grapevine red blotch disease (GRBD). Future climatic conditions are creating a more favorable environment for the proliferation of most virus-insect vectors, so the spread of virus-related diseases is expected to increase in most wine-growing regions. However, the impact of climate change on the evolution of fungal disease pressure will be variable and depending on region and pathogen, with mildews remaining certainly the major phytosanitary threat in most regions because their development rate is to a large extent temperature-driven. This paper aims to provide a review of published literature on most important grapevine fungal and viral pathogens and their impact on grape berry physiology and quality. Our overview of the published literature highlights gaps in our understanding of plant-pathogen interactions, which are valuable for conceiving future research programs dealing with the different pathogens and their impacts on grapevine berry quality and metabolism.

## Introduction

The European grapevine *Vitis vinifera* L., by far the main *Vitis* species used for wine production in the world, is host of a multitude of biotic adveristies from insects and fungi to viruses and bacteria. Downy and powdery mildew (DM and PM) are the major fungal pathogens in most wine-growing regions worldwide. Because these two pathogens were accidentally imported into Europe from North America rather recently, at the end of the 19th century, their host, the European grapevine, did not co-evolve with them and consequently does not possess natural resistances against them. Thus, in order to guarantee sustainable yield and fruit quality, viticulture depends on relatively high amounts and frequency of pesticide application, compared to other agricultural crops. Because the grapevine has been propagated vegetatively for thousands of years, it is host of a very high number (currently >80) of graft- and vector-transmitted viral diseases that can cause important economic losses in all wine-growing regions worldwide, with the most important ones being grapevine fanleaf disease (GFLD), grapevine leafroll disease (GLRD), and the recently characterized grapevine red blotch disease (GRBD; Maliogka et al., 2015; Bragard et al., 2019; Fuchs, 2020).

The effects of global climate change on disease pressure are not unambiguous. Some models predict a decreasing fungal disease pressure and, consequently, as predicted by Zito et al. (2018) for PM and DM in the region of Burgundy, mainly due to lower precipitations during the growing season. Other modelling approaches show that the increase in temperature advances the outbreak time of diseases, such as DM, leading to more severe infections and more infection cycles, due to the polycyclic nature of the pathogen (Francesca et al., 2006; Bove et al., 2020). The higher temperature during the months of May and June create also a more favorable environment for mildew development, counterbalancing the effects of precipitation reductions, which alone would have diminished the disease pressure (Salinari et al., 2007). In any case, at the global level, mildews will very likely remain the major phytosanitary threat under future climatic conditions (Bois et al., 2017). In this context, the increasingly widespread use of single-site fungicides to control DM accelerated the development of *P. viticola* strain with resistance to most of the fungicide classes (Massi et al., 2021). The predicted increase in temperature caused by global warming is already leading to advances of the development of the grape berry moth whose larvae feed on ripening grape berries, thereby providing “entry-gates” for *B. cinerea* infection (Reineke and Thiéry, 2016; Santos et al., 2020). This could consequently result in increasing berry mold infections if pest management strategies are not adapted. As for DM, rainfall and relative humidity are key factors for the onset of gray mold (Molitor et al., 2016). The increasing risk of mold infection could thus be counterbalanced by decreasing precipitations in some regions. Higher temperatures will also favor the development of insect vectors of bacterial and viral diseases and thus augments the spread of viral diseases in most growing regions (Bois et al., 2017). A better understanding of pathogen-host interactions is of upmost importance for elaborating efficient disease-management strategies in order to guarantee high quality and sustainable wine production in an evolving environment.

**Figure 1:**
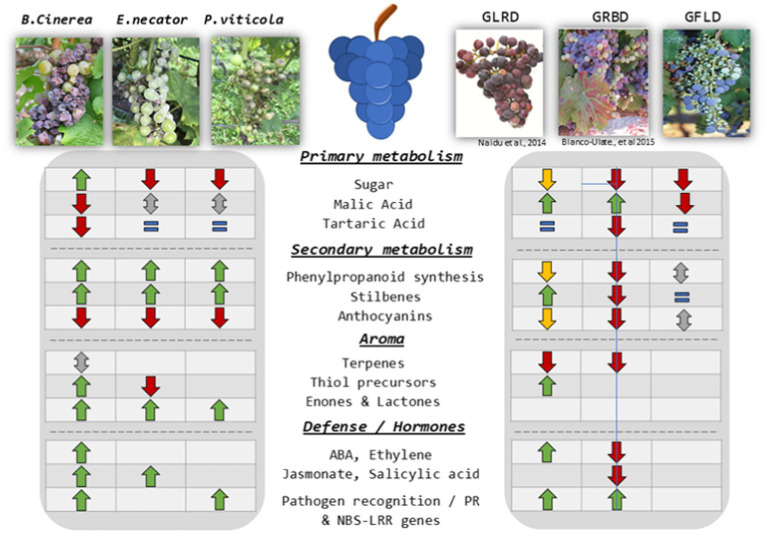
General overview of most important metabolites and mechanisms modulated by the reviewed pathogens: ↓: decrease; ↑: increase; ↕: opposed results reported; ↓: decrease only in some studies likely due to sampling strategy, thus decrease putatively observed due to a delay in phenology; =: no differences.

The lifecycle of the most common fungal diseases is well-characterized, as is their negative impact on grapevine physiology; yet, for most diseases, the molecular mechanisms that underpin the deterioration of berry quality remain to be elucidated. Recent advances in metabolomics led to the discovery of new odorous compounds produced either directly by pathogens or released by leaves or berries following infection (Darriet et al., 2002; Ky et al., 2012; Pons et al., 2018; de ferron et al., 2020). These compounds can be linked to deleterious effects on wine quality. However, molecular data regarding the interactions between pathogens and the berry are scarce. Nevertheless, the rapid development and improvement of omic tools continuously increase our insights into plant/berry pathogen interactions (Blanco-Ulate et al., 2015, 2017; Rienth et al., 2019b; Ghaffari et al., 2020; Toffolatti et al., 2020; Pimentel et al., 2021). The first part of this review aims to summarize the most important and recent studies dealing with the effects of gray mold, PM, and DM on berry quality and metabolism. Subsequently, we review the literature describing the effects on berry physiology of the most important virus-related diseases, such as grapevine fanleaf, grapevine leafroll, and red blotch disease (a general overview of modulated metabolites is provided in Figure 1 and a more detailed summary of modulated compounds and transcripts in Supplementary Table 1). Although a major attention was paid on the effects on primary and secondary metabolism, we did not review the general biosynthetic pathways because this would have gone beyond the scope of the paper. For more detailed description of biosynthetic pathways in the grape berry we invite the reader to consult recent reviews on berry development and physiology (Conde et al., 2007; Kuhn et al., 2013) as well as primary (Burbidge et al., 2021; Walker et al., 2021) and secondary metabolism (Azuma, 2018; Lin et al., 2019; Rienth et al., 2021).

## Botrytis Cinerea

*Botrytis cinerea* is among the most relevant berry-infecting fungal pathogens and is responsible for important reductions in yield and quality of grapes worldwide. *Botrytis cinerea* is responsible for both the so-called prejudicial gray mold and noble rot that will both be discussed in subsequent sections. Whether *B. cinerea* infection cause noble rot or gray mold is principally dependent on weather post-infection. Wet or humid conditions lead berries infected with *B. cinerea* to develop gray mold, whereas the development of noble rot requires moist nights and foggy mornings with dry and warm days.

### Noble Rot

Noble rot is used to produce highly valued sweet dessert wines, such as Sauternes, Tokaji Aszu, and Amarone. The positive effects of noble rot are not only due to the concentration of sugar and aroma components through *B. cinerea*-induced water loss of the berry, but also from an enhanced synthesis of aromatic metabolites and their precursors, such as odorous lactones (γ and δ lactones) and their precursors (Miklósy and Kerényi, 2004; Tosi et al., 2012; Stamatopoulos et al., 2014; Lopez Pinar et al., 2017a,b; Stamatopoulos et al., 2018). In particular, this has been shown for S-conjugates of glutathione and cysteine, the precursors of varietal thiols reminiscent of citrus, grapefruit/passionfruit, or boxwood aroma. Their concentration can increase more than 100-fold upon *B. cinerea* infection (Sarrazin et al., 2007; Bailly et al., 2009; Thibon et al., 2009, 2011). The concomitant presence of S-conjugates of glutathione and cysteine in musts from infected berries as well as in botrytized berries highlights an activation of the glutathione biosynthetic pathways, a known strategy of plants as a general response to biotic (pathogenic attack) or abiotic stresses (injury, oxidative stress etc.; Hasanuzzaman et al., 2017). This has also been shown for grapevine leaves and berries exposed to cold and heat shock as well as UV irradiation (Kobayashi et al., 2011). Biosynthesis of S-conjugated amino acids and subsequent metabolites results from an increased release of reactive and toxic aldehydes, such as trans-2-hexenal reacting with glutathione to form glutathione S-conjugates, such as S-3-(hexan-1-ol)-glutathione, which in turn will be metabolized into S-conjugate cysteine and ultimately cleaved into sulfanylalcohols during fermentation by ß-lyase activity of *Saccharomyces cerevisiae* (Tominaga et al., 1998).

Other odorous volatile compounds, such as lactones (γ and δ lactones), furanone, methional, and phenylacetaldehyde, have been found in botrytized berries (Kikuchi et al., 1983; Miklósy and Kerényi, 2004; Tosi et al., 2012; Stamatopoulos et al., 2014; Lopez Pinar et al., 2017a,b; Stamatopoulos et al., 2018). These compounds can contribute in a desirable way to the flavor of sweet wines produced from noble rot-infected grapes (Schreier et al., 1976; Sarrazin et al., 2007; Tosi et al., 2012). Organic acids, such as tartaric and malic, are metabolized by the fungi and wines from botrytized berries have generally lower total acidity (Shimazu et al., 1984).

### Gray Mold

Gray mold can be very detrimental to fruit and wine quality due to the degradation of a number of grape berry components (Pallotta et al., 1998). In particular, phenolic compounds (anthocyanins, hydroxycinnamic acids, and flavanols) are oxidized by the polyphenol oxidase (laccase) activity of *B. cinerea* (Dubernet et al., 1977), which leads to quinones, that are highly reactive with glutathione and volatile odorous thiols. Ky et al. (2012) showed that all phenolic compounds (anthocyanins and proanthocyanidin monomers, dimers and trimers) decreased drastically in gray mold-infected grape skins as well as the mean degree of polymerization of the proanthocyanidin polymeric fraction.

Some aromatic components, like monoterpenes that play a major role in the aromas of Muscat grape cultivars and wines, are transformed into less odorous compounds (Boidron, 1978; Bock et al., 1986). Ethyl esters of fatty acids, which contribute to the fermentation aromas of wine, are hydrolyzed by the esterase activity of *B. cinerea* (Dubourdieu et al., 1983). Moreover, the development of gray mold, associated with abundant sporulation, as well as the presence of spores in the must, leads to the production of mushroom and earthy off-odors in the resulting wines (La Guerche et al., 2006; Lopez Pinar et al., 2017a,b). Compounds responsible for such off-odors have been identified as 1-octen-3-one, 1-octen-3-ol, 2-heptanol, and 2-octen-1-ol with mushroom notes, or 2-methylisoborneol with strong earthy notes. Sometimes, a certain proportion of botrytized grapes (< 1–5%) are co-contaminated with other saprophytic fungi belonging to the genus *Penicillium* spp., particularly *P. expansum*, as well as *Mucor* spp., *Trichotecium* spp., *Cladosporium* spp., *Aureobasidium* spp., *Alternaria* spp., etc., which may develop inside the grape clusters, resulting in secondary rot with yellow, green, or pink shades. Their presence is generally favored by their ability to grow at lower temperatures than *B. cinerea* (10–15°C). In this context, other off-odors may be detected in wines, associated with the formation of potent, malodorous compounds, particularly caused by the presence of *Penicillium* spp., in particular *Penicillium expansum*, and its production of (−)-geosmin (Darriet et al., 2000; La Guerche et al., 2006, 2007; Steel et al., 2013; Behr et al., 2014).

### Berry Transcriptomic Modulations by *B. cinerea*

The four most recent whole-genome transcriptomic studies on *B. cinerea*-infected berries used either Microarrays (Agudelo-Romero et al., 2015; Kelloniemi et al., 2015) or RNA-seq (Blanco-Ulate et al., 2015; Lovato et al., 2019a,b) to detect differentially expressed genes upon *B. infection*. Varieties, sampling- and infection protocols vary in all studies, making it difficult to draw general conclusions. Agudelo-Romero et al. (2015) and Blanco-Ulate et al. (2015) investigate gray mold infection on Semillon berries sampled at maturity (23.9°Brix; Blanco-Ulate et al., 2015) and Trincadeira at two different stages of berry development (green berries: EL 33 and at véraison: EL 35; Agudelo-Romero et al., 2015). Both studies draw samples directly *in situ*. Kelloniemi et al. (2015) and Lovato et al. (2019b) studied noble rot on post-harvest-infected berries from Marselan berries cut at version and maturity (Kelloniemi et al., 2015) and on Muller-Thurgau and Garganega (Lovato et al., 2019b). Furthermore, the latter authors carried out a meta-analysis of transcriptomic data from all aforementioned studies on *B. cinerea*-infected grapes. They could identify only 17 commonly upregulated transcripts in all varieties and stages that were specific to *B. cinera* infection. When looking only at either gray mold or noble rot and only after véraison, commonly upregulated genes were found to be higher: 129 and 173, which seems still relatively low in comparison with other biotic (Rienth et al., 2019a) and abiotic stress (Rienth et al., 2014b; Lecourieux et al., 2017) transcriptomic studies. Obviously, this relatively low number of commonly regulated genes can, to some degree, be explained by the fact that gray mold and noble rot infections were compared from different varieties. However, it is very likely that the different, not very precisely defined and characterized berry sampling stages prevent the detection of more commonly genes modulated specifically by *B. cinerea*. It has been shown that a unprecise definition of berry sampling stages can biases biotic and abiotic stress to a high extent, mainly when studies include the stage of véraison (which is considered when 50% of berries change color), where a large transcriptomic reprogramming occurs (Terrier et al., 2005; Rienth et al., 2014a) within 24h from green to soft (and colored) berries. Thus mixing green and colored berries from a cluster at véraison can introduce unquantifiable biases that could cover important transcriptomic regulations (Carbonell-Bejerano et al., 2016; Shahood et al., 2020).

Nevertheless when looking at different metabolic pathways one can summarize that in all studies, *B. cinerea* caused an induction of secondary metabolism. Notably, the phenylpropanoid pathway was generally highly activated, as indicated by the upregulation of *R2R3-MYB*, *VviMYB5a/b* transcription factors (TFs), known as key regulators of phenylpropanoids, and the downstream key genes involved in the production of flavonoids, such as chalcones, flavonols, and anthocyanins, procyanidins, lignin, and lignans (Blanco-Ulate et al., 2015; Lovato et al., 2019a,b).

In particular, the biosynthesis of stilbenes, major defense compounds (Gindro et al., 2012a), is highly activated upon *B. cinerea* infection, as highlighted by the induction of *VvMYB14*, *VvSTSs* and *PAL* expressions in samples of Lovato et al. (2019a,b) which was similar to that of berries sampled at the véraison stage by Kelloniemi et al. (2015). Interestingly, in the white cultivar Semillon, noble rot caused by *B. cinerea* induced the expression of transcriptional regulators normally expressed in the skins of red cultivars, including five *R2R3-MYBs* that regulate stilbene (*VvMYB14,VvMYB15*), proanthocyanidin (*VvMYBPA1*), and phenylpropanoid (*VvMYBC2-L1, VvMYB4a*) metabolism, as well as other potential regulators of ripening, such as *VvNAC33, VviNAC60*, a zinc-finger transcription factor, a MYB transcription factor, and an *AP2/ERF* transcription factor (Blanco-Ulate et al., 2015).

Transcriptomic data regarding volatile compounds biosynthesis highlight a cultivar-dependent response. For example, Blanco-Ulate et al. (2015) observed a general upregulation of several terpene synthases, *VvTPS*, in Sémillon botrytized berries, which was, however, not confirmed in Trincadeira (Agudelo-Romero et al., 2015) or Muller-Thurgau and Garganega (Lovato et al., 2019b), where terpene biosynthetic genes were generally downregulated by the infection. A further, more general physiological response of berry metabolism upon *B. cinerea* infection is the upregulation of oxidative stress-related transcripts, abscisic acid, ethylene, jasmonate, and salicylate pathways, and genes encoding resistance (PR-genes; Agudelo-Romero et al., 2015; Coelho et al., 2019; Lovato et al., 2019b). Coelho et al. (2019) compared the hormonal response in *Botrytis*-infected berries of susceptible (Trincadeira) and resistant (Syrah) varieties and showed that high basal levels of salicylic acid (SA) and indoleacetic acid (IAA) at an early stage of ripening, together with activated SA and IAA metabolism and signaling seem to be important in providing a fast defense response leading to grape tolerance against *B. cinerea*.

Interestingly, a group of sugar transporter (*SWEETs*) seems to play an important role during *B. cinerea* infections as shown for the first time by Chong et al. (2014), who demonstrated an involvement of the grapevine *SWEET transporter 4* (*VvSWEET4*). Moreover, they further showed that *VvSWEET4* is a glucose transporter localized in the plasma membrane, which is upregulated by inducers of reactive oxygen species and virulence factors from necrotizing pathogens. This led authors to the hypothesis that stimulation of expression of a developmentally regulated glucose uniporter by reactive oxygen species production and extensive cell death after necrotrophic fungal infection could facilitate sugar acquisition from plant cells by the pathogen. Later, Breia et al. (2020b) highlighted the role of several other *VvSWEETs* in grapevine berries upon pathogen infections. Notably the mono- and disaccharide transporter *VvSWEET7* was strongly upregulated during *B. cinerea* infection of grape berries. This induction may be caused by the pathogen itself to promote leakage of sugars into the apoplastic space for nutrition, or, as a defense-related process to improve sugar remobilization which can trigger signaling cascades that activate plant defense mechanisms. The same authors showed as well that grapevine’s sucrose transporter *Early-Response to Dehydration six-like 13* (*VvERD6l13*) was strongly upregulated in response to *B. cinerea* but as well by *E. necator* infection (Breia et al., 2020a).

As mentioned above, several studies report increased thiols, which correlate with the detoxification pathway and increased lactone content in wines and musts produced from *B. cinerea*-infected grapes. Together with the upregulation of the phenylpropanoid, anthocyanin, stilbene, and terpene pathways, and with the induction of defense phytohormones pathway, this highlights a deep metabolic reprogramming as a plant defense mechanism against *B. cinerea* infection. However, transcriptomic studies are unambiguous regarding volatile synthesis. More studies including more varieties and precise definitions of berry sampling and infection protocols are required in the future to better characterize stage and variety dependent responses to *B. cinerea* which could also give more insight on genotypic plasticity of tolerance to infection of some varieties.

## Downy Mildew – *Plasmopara Viticola*

Downy mildew infections on leaves can cause important losses of leaf surface, and thus negatively affect carbon status at the vine level. This leads to ripening delay, slacking sugar accumulation, and malic acid respiration in berries. Therefore, grapes from vines with high infections of *P. viticola* have in general lower total soluble solids, less juice color, and higher acidity compared to fruits from healthy grapevines. DM can also infect berries at early stages, as well as the pre-bloom flowers, pedicel, and rachis (Kennelly et al., 2005). Mature berry infection is strongly restricted by ontogenic resistance due to the progressive modifications of stomata into lenticel at véraison (Gindro et al., 2012b). After véraison, sporulation can still be seen on pedicel and rachis with little to no consequence for berry quality. Probably, because of this almost-absence of DM infection after fruit set, few studies were conducted to understand the effect of *P viticola* on berries. To understand the resistance mechanism of different cultivars, Gindro et al. (2012a) analyzed stilbene content in grape clusters of susceptible cultivars, such as Chasselas and Merlot, and resistant ones, such as Solaris and the hybrid 2091, after *P. viticola* infection. At the inflorescence stage (BBCH 53), in non inoculated conditions, susceptible cultivars presented a high basal content of piceid compared to the resistant ones. A shift in stilbene content occurred after inoculation for all cultivars. Upon infection susceptible cultivars showed a high accumulation of piceid while other stilbenes remained at lower concentrations. For Solaris, a higher accumulation of piceid, resveratrol, δ- and ε-viniferins, and pterostilbene compared to the control is observed. The 2091 hybrid showed a high accumulation of piceid and resveratrol and δ-viniferin and a lower accumulation of ε-viniferin and pterostilbene. At later stage (end of flowering and berry pea-sized stages), a gradual diminution of stilbene content was observed, which was longer in susceptible cultivars, especially for Chasselas, with a high accumulation of resveratrol at the end of flowering that would later be metabolized at the berry pea-sized stage. Beside the stilbene-related plant defense response, DM berry infections modify the grape aroma profile. These modifications are associated with increased concentrations of lactones in wines, such as γ-octalactone, γ-nonalactone, and γ-decalactone, as well as a significant proportion of volatile compounds issued from unsaturated fatty acid degradation, such as (Z)-1,5-heptadien-3-one and (Z)-1,5-octadien-3-one (geranium leaves descriptors; Pons et al., 2018). Furthermore, 3-methyl-2,4-nonanedione (MND), a powerful β-diketone identified in prematurely aged red wines marked with an intense prune flavor, is more abundant in wines produced from grapes that include diseased berries. Finally, Merlot wines produced with increased levels of *P. viticola*-infected berries presented a higher intensity of the “cooked fruit” character and sometimes green nuances (Pons et al., 2018). Recently, Poitou et al. (2021) evidenced the impact of Methyl salicylate (MeSA), which induces plant defense resistance and is an odorous volatile compound presenting green nuances in wine. The latter authors could show that *P. viticola*-infected grapes had higher concentration of MeSA than healthy ones and showed that this aroma compound contributes in the expression of fresh green aromatic nuances in red wines, e.g., “pharmaceutical,” “camphor,” or “menthol” aromas. With the exception of stilbene-related defense mechanisms and lactone biosynthesis, the impact of *P. viticola* on berries remains elusive and requires deeper investigation, in particular from a molecular point of view.

## Powdery Mildew – *Erysiphe Necator*

Together with DM, PM is the most important fungal disease infecting mainly grapevine leaves. Berry infections can occur during the green growth phase and are particularly detrimental to quality when they occur around fruit set. Berry infections during ripening are reported to occur until soluble solids levels reach 8°Brix (Gadoury et al., 2003). As for DM, foliar infections can cause important losses of photosynthetic leaf area, which leads mainly to a delay in ripening, thus lower sugar concentration and higher acidity. When grape berries are directly infected with PM, they mostly show similar soluble solids content as healthy grapes but with significantly lower yields (Pool et al., 1984; Gadoury et al., 2001; Stummer et al., 2003, 2005; Calonnec et al., 2004; Pimentel et al., 2021). Contradictory results are reported concerning titratable acidity of infected berries, with some authors reporting an increase (Calonnec et al., 2004) and others a decrease (Lopez Pinar et al., 2016). Anthocyanin concentration was lower in Cabernet Sauvignon (Calonnec et al., 2004) and Sangiovese (Piermattei et al., 1999) in wines produced with PM-contaminated berries. Total phenolics, hydroxycinnamates, and flavonoids were higher in wines from infected grapes from the white cultivar Chardonnay (Stummer et al., 2003, 2005).

Studies about effects on aroma compounds are scarce. Calonnec et al. (2004) report a decrease of the thiol 3- sulfanylhexan-1-ol (3SH) in Sauvignon blanc wines and high concentrations of lactones (γ-butyrolactone, γ-dodecalactone, and γ-decalactone) similarly to what also observed in Riesling when berries were affected with PM (Lopez Pinar et al., 2017a,b).

Another important aspect of PM development on grapes concerns the presence of 1-octen-3-one (mushroom-like notes), (*Z*)-1,5-octadien-3-one (geranium leaf-like notes), phenylacetic acid (honey notes), and (R)-carvone (spearmint notes). The enone aroma-related compounds mentioned above generally diminish and sometimes disappear during winemaking, a phenomena related to the enzymatic reduction of main off-odors [1-octen-3-one, (*Z*)-1,5-octadien-3-one] to less odorant compounds [3-octanone, (*Z*)-5-octen-3-one] by enone reductase of *Saccharomyces cerevisiae* (Wanner and Tressl, 1998; Darriet et al., 2002).

Transcriptomic studies on PM infection of grapevine berries as scarce. Up to our knowledge, the only comprehensive metabolomic and whole-genome transcriptomic study on PM-infected berries was conducted on Grenache berries on developmental stages EL32 (green berry at bunch closer) and EL35 (véraison) by Pimentel et al. (2021). They report a strong indication of defensive mechanisms upon PM infection indicated by higher levels of jasmonates and salicylic acid together with the secretion of effectors related to effector-triggered susceptibility, such as PR1 genes and Enhanced disease susceptibility 1 (EDS 1). PM infection lead as well to an upregulation of carbohydrate-active enzymes, fatty acid and nitrogen uptake and the increase of metabolites, such as gallic, eicosanoic and docosanoic acids, and resveratrol, which could serve as potential metabolic biomarkers, that could be used to monitor the early stages of the infection (Pimentel et al., 2021). Furthermore, PM infection induced an activation of key phenylpropanoid pathway genes (*PAL, C4H, 4CL, CHS, F3H*) and accumulation of catechins, resveratrol and an overexpression of the respective transcripts, such as *LAR/ANS* and *STS*s. Anthocyanins transcripts (*F3’5’H, F3’H,UFGT*) were also found to be upregulated in early stages of berry development, which goes along with previous studies on Chardonnay (Stummer et al., 2003, 2005). These transcripts were particularly upregulated in the green berry (EL32) and correlated with higher anthocyanin content. This was however not significant anymore at EL35. Since anthocyanin accumulation starts at later stages after véraison a clear conclusion on the impact on anthocyanins at maturity cannot be drawn on the basis of these results.

Infection of berries with secondary fungi can be responsible not only for off flavors in grape and wine but also for high concentration of phytotoxin, such as ochratoxin A (OTA) or fumonisin B2 (FB2).

Indeed, in an interesting study, it was seen that Negroamaro berries, infected with PM were significantly more susceptible to both *Aspergillus niger* and *Aspergillus carbonarius* colonization which produce FB2 and OTA (Cozzi et al., 2013).

## Virus Infections

The present section will summarize the impact on berry composition of the most important viral diseases, GLRD, GFLD, as well as the recently discovered and described Grapevine red blotch and Pinot Gris virus (Sudarshana et al., 2015; Blanco-Ulate et al., 2017; Adiputra et al., 2018; Reynard et al., 2018; Cieniewicz et al., 2020).

In general, symptoms of virus infections in plants strongly vary according to genotype and, up to a great extent, to pedoclimatic conditions (Cretazzo et al., 2010). The symptoms differ as well between plant tissues, sometimes triggering completely opposite metabolic responses in berries (Vega et al., 2011) or leaves as was shown for GLRD (Perrone et al., 2017).

### Grapevine Leafroll Disease

Grapevine leafroll disease is considered the most widespread and devastating virus-associated disease. So far, nine serologically distinct virus types from the *Closteroviridae* family were associated with GLRD, named grapevine leafroll associated virus (GLRaV) types 1–9, with the most widespread ones being GLRaV-1 and GLRaV-3 (Maree et al., 2013; Velasco et al., 2014). GLRD causes important reductions in yield, vigor, and longevity of vines. It also delays fruit ripening, reduces sugar accumulation, and impairs fruit pigmentation (Guidoni et al., 1997; Maree et al., 2013; Naidu et al., 2014, 2015; Alabi et al., 2016). Decreases in anthocyanin and total flavonoid concentrations due to GLRD infections were related to the downregulation of anthocyanin-related transcripts, such as *VvUFGT*, *VvMYABA1,* and other phenylpropanoid genes, such as *VvCHS*, *VvFLS1,* and *VvMYAPA1* (Vega et al., 2011; Vondras et al., 2021). Vega et al. (2011) also observed a viral repression of sugar transporters, which translated to lower sugar concentration in grapes. Interestingly, a study using a different berry sampling approach, which accounts for potential phenological shifts and intracluster berry heterogeneity, has demonstrated that the downregulation of genes belonging to primary and secondary metabolite pathways was mainly due to the berry phenological delay induced by GLRD infection and not to a direct effect of the viral infection (Rienth et al., 2019b; Ghaffari et al., 2020). In a very comprehensive study on Carbernet Franc over 2years on different rootstocks, Vondras et al. (2021) observed a rootstock specific transcriptomic response of berries from GLRaV-infected vines. Latter authors observed the modulation of genes related to pathogen detection, for example NBS-LRR genes that confer resistance to powdery and downy mildew (DM) in grapevine (Riaz et al., 2011; Zini et al., 2019), as well as abscisic acid (ABA) signaling, phenylpropanoid biosynthesis, and cytoskeleton remodeling similar to previous studies of Vega et al. (2011) and Ghaffari et al. (2020). Interestingly the increase of ABA abundance in GLRD-infected berries (Vondras et al., 2021) was different than that observed for red blotch virus-infected berries, in which ABA abundance and *NCED* expression decrease in infected berries after véraison (Blanco-Ulate et al., 2017).

In leaves, some studies report a similar upregulation of defense-related genes and a concomitant accumulation of phenylpropanoids, such as resveratrol, which led to an enhanced resistance to downy mildew (Repetto et al., 2012). This highly interesting observation was somehow confirmed in a very elegant experiment, where authors transmitted GFLaV and grapevine rupestris stem pitting-associated virus (GRSPaV), by *in vitro*-grafting, to Nebbiolo and Chardonnay. Upon subsequent downy and powdery mildew infection, GFLV-infected plants showed a reduction in severity of the diseases caused by powdery and downy mildews in comparison to virus-free plants, which highlights a potential upregulation of plant innate immunity by GLRD infection (Gilardi et al., 2020).

### Grapevine Red Blotch Disease

Grapevine red blotch disease is caused by the Grapevine red blotch-associated virus (GRBaV) and was discovered in 2008 in California. GRBD has recently become a major economic problem for the wine industry in many growing regions (Sudarshana et al., 2015; Adiputra et al., 2018; Reynard et al., 2018). GRBaV infections result in the appearance of red patches on the leaf blades, veins, and petioles in red grape varieties, whereas in white grape varieties, they cause irregular chlorotic areas on the leaf blades. Detrimental effects of red blotch disease are similar to GLRD. GRBaV affects berry physiology, causing uneven ripening, higher titratable acidity, and lower anthocyanin and sugar content (Girardello et al., 2019, 2020; Rumbaugh et al., 2021) putatively due to an impairment of carbon import into the berry (Martínez-Lüscher et al., 2019). Rumbaugh et al. (2021) observed a general decrease of fruity aroma compounds in Cabernet Sauvignon, mostly linked to a reduction of monoterpenes, such as limonene, ß-myrcene, α-terpinene, geranial, and p-cymene. Higher titratable acidity has been attributed to lower acid respiration as shown by Pereira et al. (2021), who found 56% higher malic acid and lower tartaric acid and phenolic compounds, such as the flavan-3-ols, catechin, epicatechin and the flavonol quercetin-glucoside, in Cabernet Sauvignon infected with GRBaV.

Using RNA-seq for differential gene expression analysis, Blanco-Ulate et al. (2017) associated the GRBD-induced deterioration of grape quality with a downregulation of key genes of the phenylpropanoid pathway, which confirms aforementioned phenotypic observation. Furthermore, Blanco-Ulate et al. (2017) showed that GRBD disturbs berry development and induces stress responses by altering transcription factors (e.g., *VviNACs*, *VviMYBs*, and *VviAP2*-*ERFs*) and phytohormone networks causing an inhibition of the ripening process thus reducing color, flavor, and aroma compounds in berries.

### Grapevine Fanleaf Disease

Grapevine fanleaf disease is one of the oldest known viral diseases of grapevines and has been found in all wine-growing regions around the world (Raski et al., 1983; Silva et al., 2017). GFLD has been reported to cause significant economic losses by reducing grape yield due to reduction of both cluster weight and berry weight, shortening the longevity of vines, and affecting fruit quality by decreasing the sugar content and titratable acidity (Raski et al., 1983). More detailed studies focusing on berry physiology and quality are not very numerous and often ambiguous. It has been shown that GFLD can also affect the anthocyanin levels in a cultivar-dependent manner. In the variety Manto Negro, GFLD reduced the anthocyanin level (Cretazzo et al., 2010), while in Schioppettino, an increase in anthocyanin content is observed and the relative proportions between di- and tri-hydroxylated or -methylated derivatives of anthocyanins seem to be affected *via* the upregulation of *VviF3H1* and *VviF3’H5* and downregulation of *VviF3H* (Rupnik-Cigoj et al., 2018). Further studies on berries from GFLD-infected vines, including several developmental stages and cultivars, are utterly needed to better understand the effect on fruit quality of this widespread pathogen.

### New Emerging Virus-Associated Diseases

Recently, several emerging viruses have been described (Cieniewicz et al., 2020), with the Pinot Gris Virus (Giampetruzzi et al., 2012) being the most threatening one. It is present in most wine-growing regions from North (Al Rwahnih et al., 2016) and South America (Debat et al., 2020) to almost all the European countries, such as Italy (Gentili et al., 2017), Germany (Messmer et al., 2021), Spain (Ruiz-García and Olmos, 2017), and France (Renault-Spilmont et al., 2018). Although leaf symptoms are well-characterized, its impact on berry physiology has not yet been characterized in detail. The most important detrimental impact of Pinot Gris virus in viticulture is certainly the great reduction in yield, which can range between 66 and 85%, as shown for Glera and Pinot Noir; however, it causes no significant alteration of fruit quality (Bertazzon et al., 2017).

## Conclusions

The consequences of the most important fungal and viral diseases on grapevine performance, longevity, yield, and berry and wine quality are mostly well known. However, fundamental molecular studies, aiming to characterize the underpinning mechanisms involved in berry-pathogen interactions are scarce.

The most studied fungal pathogen, due to its multiplicity of host plants and fruits and its partly beneficial effects in the production of noble rot wines, is *B. cinerea*. For powdery and downy mildew and their rather indirect impact on berry development and quality, published studies focus almost entirely on the leaves’ transcriptome and metabolome without considering berry development and metabolism. More molecular studies are therefore needed to gain more insight into the berry-pathogen interactions and to better characterize the negative effect of fungal pathogens on leaves as well as on berries. Furthermore, it would also be useful that future studies on berry-pathogen interactions focus more on a precise definition of the berry developmental stage and account for post-véraison berry heterogeneity (Carbonell-Bejerano et al., 2016; Rienth et al., 2021). This would improve comparability and thus yield better insights in molecular berry-pathogen interactions.

It would also be interesting from a growers’ and scientific point of view to conduct more studies investigating the combined effects of fungal pathogens and their corresponding conventional or organic fungicides on berry composition and metabolism, as investigated by Rantsiou et al. (2020).

A growing concern in worldwide viticulture is grapevine trunk diseases, due to a lack of studies on them. Common consequences on berry quality seem to be lower sugars and phenolic compounds, such as catechin, epicatechin, and anthocyanins (Lorrain et al., 2012; Fontaine et al., 2016). However, detailed physiological and omic studies are missing thus far. Viral diseases cause a global perturbation of the plant physiology with strong effects on primary and secondary metabolism that leads to interference with the ripening of berries. Characterization of the broad effect of viral disease is still in its infancy, but an increasing number of studies is being published using state-of-the-art molecular tools to provide valuable knowledge on plant/fruit-pathogen interactions. However, more coordinated efforts at genomic, transcriptomic, and metabolomic levels, in particular including epigenetics, should be deployed to understand and better characterize the differences among *V. vinifera* cultivars and their different responses to diseases. Furthermore, integrative studies comprising multiple simultaneous infections with different viral and fungal pathogens (Gilardi et al., 2020), even with abiotic stresses and multiple genotypes, should be considered in the future to better anticipate disease impact in a climate change scenario. A deeper understanding of defense response mechanisms in various *V. vinifera* as well as other *Vitis* spp. could help identify new resistance traits essential for improving breeding programs as well as for the development of biopesticides and biostimulants.

## Author Contributions

MR, SC, and PD devised the main body and structure and content of the manuscript. CS, CBu, CBo, NV, RW, and FF provided valuable ideas and corrections and triggered fruitful discussions *via* helpful comments. All authors contributed to the article and approved the submitted version.

## Funding

The postdoc of NV was financed by the Swiss National Science Foundation (SNF) Project Grant IZCOZ0_189896.

## Conflict of Interest

The authors declare that the research was conducted in the absence of any commercial or financial relationships that could be construed as a potential conflict of interest.

## Publisher’s Note

All claims expressed in this article are solely those of the authors and do not necessarily represent those of their affiliated organizations, or those of the publisher, the editors and the reviewers. Any product that may be evaluated in this article, or claim that may be made by its manufacturer, is not guaranteed or endorsed by the publisher.

## Supplementary Material

Supplementary Table 1 Selection of important metabolites, genes, and transcription factors that have been shown to be modified by different pathogens.
